# Phytochemical Screening, Antibacterial Activity and Heavy Metal Analysis of Ethnomedicinal Recipes and Their Sources Used Against Infectious Diseases

**DOI:** 10.3390/plants8110454

**Published:** 2019-10-26

**Authors:** Nasir Mahmood, Ruqia Nazir, Muslim Khan, Rashid Iqbal, Muhammad Adnan, Mohib Ullah, Hongyi Yang

**Affiliations:** 1 Key Laboratory of Saline-alkali Vegetation Ecology Restoration (Northeast Forestry University), Ministry of Education, Harbin 150040, China; nasirbabrum@yahoo.com; 2 College of Life Sciences, Northeast Forestry University, Harbin 150040, China; 3 Department of Chemistry, Kohat University of Science and Technology Kohat 26000, KPK, Pakistan; chemist352@gmail.com (R.N.); nasirnustian4@gmail.com (M.K.); Rashidkhan1@gmail.com (R.I.); 4 Department of Botanical and Environmental Sciences, Kohat University of Science and Technology, Kohat 26000, Khyber Pakhtunkhwa, Pakistan; ak4536975@gmail.com; 5 Key Laboratory of Functional Inorganic Material Chemistry, Ministry of Education (School of Chemistry and Material Science), Heilongjiang University, Harbin 150080, China; mohib3086@gmail.com

**Keywords:** phytochemicals, antibacterial activities, medicinal plants, recipes

## Abstract

Plants are a rich source of secondary metabolites that have been found to have medicinal properties. The present study was conducted to evaluate the phytochemical screening, antibacterial activities and heavy metal analysis of seven medicinal plants i.e., *Nigella sativa* (seeds), *Trigonella foenum-graecum* (seeds), *Brassica campestris* (seeds), *Pistacia integerrima* (galls), *Linum usitatissimum* (seeds), *Hyssopus officinalis* (flowers), *Ephedra vulgaris* (dry branches) and its two recipes which are used by hakims (Practitioners of local herbal medicines), against different diseases particularly respiratory tract infections. The obtained results revealed that alkaloids (30%) and flavonoids (41%) were in maximum quantity in *P. integerrima* (galls) while saponins (10.9%) were in maximum quantity in Recipe 1. The antibacterial activity was determined by the agar well disc diffusion method using methanol, ethanol, chloroform and deionized water extracts. Each plant extract was tested against one Gram-positive (*Streptococcus pneumonia*) and two Gram-negative (*Pseudomonas aeruginosa* and *Klebsiella pneumonia*) bacteria. Maximum zones of inhibition in methanol, ethanol, chloroform and aqueous extract were seen in *T. foenum-graecum* against *S. pneumonia* (20.06 ± 0.16 mm), *B. campestris* against *S. pneumonia* (22.40 ± 0.24 mm), Recipe 2 against *K. pneumonia* (20.06 ± 0.16 mm) and *N. sativa* against *S. pneumonia* (20.23 ± 0.16 mm), respectively. The concentrations of heavy metals were determined by atomic absorption spectrophotometer and showed the existence of high concentration of Iron (Fe), Lead (Pb) and Chromium (Cr). Thus, it has been found that medicinal plants individually as well as their recipes are potentially active against various diseases particularly respiratory tract infections

## 1. Introduction

Medicinal plants are indispensable components of earth as they are used to cure different infectious diseases all over the world and are a source of many effective and powerful drugs [1]. Medicinal plants contain secondary metabolites like alkaloids, flavonoids, saponins, tannins and glycosides which are used against different diseases and relieve pain [2,3]. Many studies have shown the therapeutic activities of secondary metabolites obtained from medicinal plants [4,5]. There are several manuals which have reported the ethnomedicinal properties of the plants [6,7]. Recent studies have shown the alkaloids obtained from medicinal plants were highly active against *Staphylococcus aureus* [8]. With the advancement in the study of antimicrobial resistance, phytochemicals of medicinal plants lay down the foundations for new pharmaceuticals [9,10]. Herbal medicines are mostly used because they are easily available, have low toxicity, are inexpensive and have few side effects. Doctors are usually ambivalent to advise them because of insufficient knowledge and fear about liability. Most people, particularly in rural areas, are still using traditional medicines for their common ailments due to poverty and unavailability of modern health facilities [11,12].

The flora of Pakistan due to many ecological regions with its diverse climatic and soil conditions has a large number of medicinal plants [13]. According to survey, approximately 6000 species of plants have been reported in Pakistan and 600 of them have medicinal importance, majority of which occurs in the Himalayan region [14]. Approximately 300 medicinal plants are traded and 12% of Pakistani floras are used in these medicines. More than 75% of population in this country is treated by using traditional medicines recommended by more than 50,000 traditional herbal practitioners locally called hakims. The ten most important Davakhanas or herbal manufacturers have annually consumed 200 medicinal plants and their utilization in the last two decades [14,15]. The natural resources and wealth of our country are becoming infected as a result of increased industrialization, urbanization, human population, discharge of organic hydrocarbons and inorganic heavy metals into the atmosphere [16]. It is necessary to encourage and find out plant derived medicines in Pakistan, where infectious diseases are common. Microbes can be destroyed or controlled by using these medicinal plants [17].

The mechanism of action and efficiency of herbal extracts in most cases are yet to be scientifically validated. Therefore, significant work has been carried out by researchers to focus their attention towards traditional medicines for the development of outstanding drugs used against different varieties of microbial infections. For examples, many medicinal plants such as *Nigella sativa* L., *Brassica campestris* L., *Trigonellafoenum-graecum* L., *Pistacia integerrima, Linum usitatissimum* L., *Hyssopus officinalis* L., *Ephedra vulgaris* and their two recipes (Recipe 1 and 2), which are mostly used in many regions of Khyber Pakhtunkhwa to locally treat the infectious diseases caused by bacteria without their scientific exploration [18]. Recipe 1 is the combination of four different plants like *Nigella sativa* (seeds), *Brassica campestris* (seeds), *Trigonellafoenum-graecum* (seeds) and *Pistacia integerrima* (galls) mixed with the ratio of 1:1:2:4, respectively. While, Recipe 2 is the combination of three plants i.e., *Linum usitatissimum* (seeds), *Hyssopus officinalis* (flowers) and *Ephedra vulgaris* (dry branches) mixed with a 1:1:1 ratio, respectively.

Hence, the present study has been planned to investigate the phytochemical constituents, antibacterial properties and heavy metals determination which is further studied in order to assure their uses in traditional medicines. Moreover, this study will also provide basis for the new drug discovery and the isolation of bioactive compounds, which can be used against different diseases.

## 2. Results

### 2.1. Phytochemical Determination, Antibacterial Activity and Heavy Metals Analysis of Recipe 1 and Its Individual Plants 

**Figure 1:**
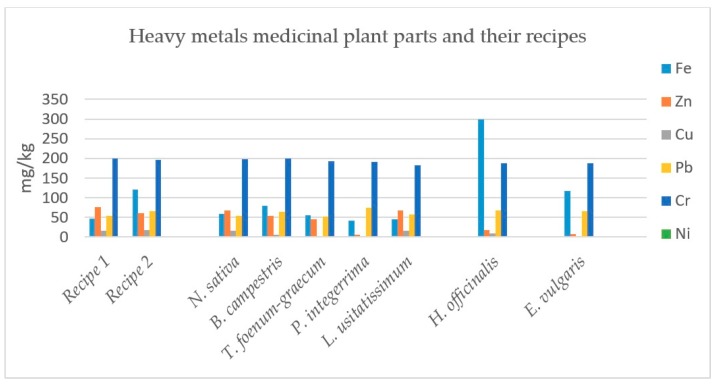
Mean concentration of heavy metals in mg/kg in medicinal plants and in their recipes. Each column represents mean value of three independent replicates and the error bars indicate standard deviation.

Recipe 1 (made of the four plants) contains 10.40% alkaloids whereas its plant parts *N. sativa* (seeds), *B. campestris* (seeds), *T. foenum-graecum* (seeds) and *P. integerrima* (galls) individually contain 1.30%, 3.20%, 3.20% and 30.0% alkaloids, respectively. Recipe 1 also contains 28.50% flavonoids whereas its plant parts *N. sativa* (seeds), *B. campestris* (seeds), *T. foenum-graecum* (seeds) and *P. integerrima* (galls) individually contain 13.00%, 10.00%, 13.80% and 41.20% flavonoids, respectively. Recipe 1 has also 10.90% saponins whereas its plant parts *N. sativa* (seeds), *B. campestris* (seeds), *T. foenum-graecum* (seeds) and *P. integerrima* (galls) individually contain 1.15%, 0.30%, 2.45% and 3.60% saponins, respectively, as shown in Table 1. All the metal constituents of the recipe 1, and its sourced plants are shown in Figure 1.

**Figure 2:**
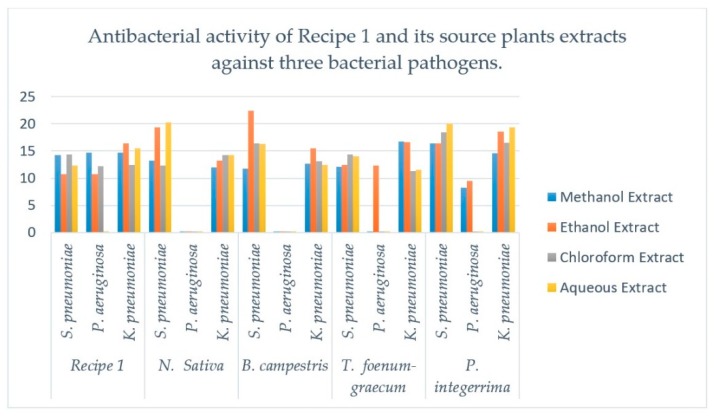
Comparative antibacterial activities of methanol, ethanol, chloroform and aqueous extracts of the *N. sativa* (seeds), *B. campestris* (seeds), *T. foenum-graecum* (seeds) and *P. integerrima* (galls) in Recipe 1. Each column represents the mean value of three independent replicates and the error bars indicate standard deviation.

The four different extracts like methanol, ethanol, chloroform and water of all these medicinal plants (used in making Recipe 1) and their recipes were used to evaluate the antibacterial activities used against three bacterial strains which are comprehensively shown in Table 2 and Figure 2. The four studied extracts of *N. sativa* shows inhibition against the bacterial strains *S. pneumonia* and *K. pneumonia* but it shows no activity against *P. aeruginosa,* respectively. Thus, by observing all of the results, it was found that the aqueous extract shows maximum zone of inhibition (20.23 mm) against *S. pneumonia* and the extract of methanol show minimum zone of inhibition against *K. pneumoniae.* The zone of inhibition of two bacterial strains was ranged from 12.03 mm to 20.23 mm. The four different extracts of *B. campestris* (seeds) were also used against three bacterial pathogens. It was noted from the results that all the four extracts show inhibition zones against two bacterial pathogens i.e., *S. pneumonia* and *K. pneumonia* but have no inhibition zone against *P. aeruginosa.* The crude extract of *B. campestris* (seeds) show maximum inhibitory effects (inhibition zone 22.40 mm) in ethanol against *S. pneumonia* and minimum effects of inhibition (inhibition zone of 11.76 mm) in methanol extract used against *S. pneumonia*. Four extracts of *T. foenum-graecum* (seeds) were used against three bacterial pathogens. It was noted from the result that methanol, chloroform and aqueous extracts have no effects of inhibition against *P. aeruginosa.* Thus, it is observed that the maximum inhibitory zone of 16.70 mm in methanol extract against *K. pneumoniae* and minimum zone of inhibition of 11.30 mm in chloroform extract against *K. pneumoniae*. The four extracts of *P. integerrima* (galls) were used against three bacterial pathogens. It was noted from the results that chloroform and aqueous extracts have no inhibition activities against *P. aeruginosa.* The maximum inhibitory zone of 20.06 mm in aqueous extract against *S. pneumoniae* and minimum inhibition zone of 8.30 mm in methanol extract against *P. aeruginosa* was observed. Four extracts of Recipe 1 were used against three bacterial pathogens. It was concluded from the results that aqueous extract has no inhibitory activities against *P. aeruginosa.* The maximum inhibitory zone of 16.46 mm in ethanol extract against *K. pneumoniae* and minimum inhibition zone of 10.70 mm in extract of methanol against *S. pneumoniae* were observed. It is well noted that Ceproflaxacene 5-µg standard well discs was used as a positive control for all the samples and dimethyl sulfoxide (DMSO) as negative control.

The results illustrated in Table 3 and Figure 1 indicate that cadmium was not detected in all the plant extracts and their recipes, while the concentration of iron was the highest at 80.43 ± 0.060 mg/kg in *Brassica campestris.* The concentration of zinc was 76.98 ± 0.019 mg/kg in Recipe 1. The concentration of copper was 16.77 ± 0.009 mg/kg for *Nigella sativa.* The maximum concentration of lead was 74.46 ± 0.165 mg/kg in *Pistacia integerrima.* The concentration of chromium was noted to be 200.07 ± 0.152 mg/kg for *Brassica campestris*. The concentration of nickel was 0.69 ± 0.014 mg/kg in case of *Nigella sativa.*

### 2.2. Phytochemical Classes Determination, Antibacterial Activity and Heavy Metal Analysis of Recipe 2 and Its Individual Plants 

Recipe 2, which is the combination of the three plants in ratio 1:1:1, contains 7.80% alkaloids while its individual plant parts *L. usitatissimum* (seeds), *H. officinalis* (flowers) and *E. vulgaris* (dry branches) contain 3.20%, 5.00% and 10.40% alkaloids, respectively. Recipe 2 contains 12.70% flavonoids while its plant parts *L. usitatissimum* (seeds), *H. officinalis* (flowers) and *E. vulgaris* (dry branches) contain 6.10%, 8.10% and 28.50% flavonoids, respectively. Recipe 2 contains 1.00% saponins while its plant parts *L. usitatissimum* (seeds), *H. officinalis* (flowers) and *E. vulgaris* (dry branches) contain 0.65%, 1.05% and 2.20% saponins, respectively as shown in Table 1.

**Figure 3:**
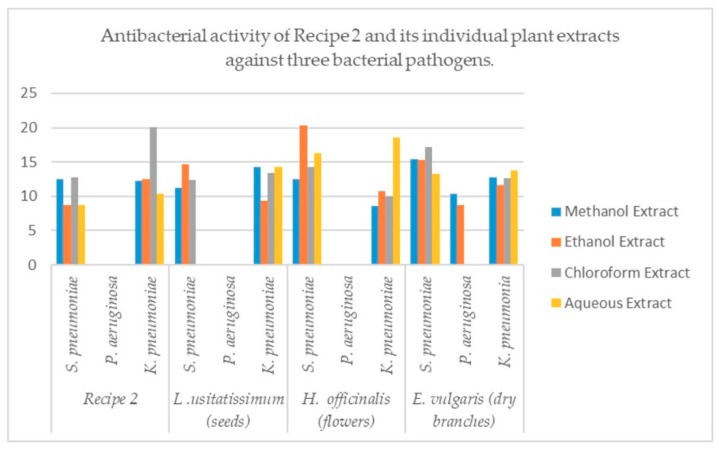
Comparative antibacterial activities of methanol, ethanol, chloroform and aqueous extracts of the *L. usitatissimum* (seeds), *H. officinalis* (flowers) and *E. vulgaris* (dry branches) and their Recipe 2. Each column represents the mean value of three independent replicates and the error bars indicate standard deviation.

The four different extracts like methanol, ethanol, chloroform and water of *L. usitatissimum* (seeds) were used against three bacterial strains. It observed from results that ethanol extract shows maximum zone of inhibition (14.60 mm) against *S. pneumoniae.* The minimum inhibition zone of 9.30 mm, was observed in ethanol extract against *K. pneumoniae*. All the four extracts show no inhibition zones against *P. aeruginosa* and *S. pneumoniae*. The four extracts of *H. officinalis* (flowers) were used against three bacterial strains. It was observed from results that ethanol extract shows maximum zone of inhibition (20.30 mm) against *S. pneumoniae.* The minimum zone of inhibition (8.56 mm) was observed in methanol extract against *K. pneumoniae*. All the four extracts show no inhibition zones against *P. aeruginosa*. Four extracts of *Ephedra vulgaris* (dry branches) were used against three bacterial pathogens. It was noted from the results that chloroform and aqueous extracts have no effects of inhibition against *P. aeruginosa.* The maximum inhibitory effects (17.16 mm inhibition zone) in chloroform extract against *S. pneumoniae* and minimum inhibition activities (8.70 mm zone of inhibition) in the extract of ethanol against *P. aeruginosa* were observed. Four extracts of Recipe 2 were used against three bacterial strains. It was observed from results that chloroform extract shows maximum zone 20.30 mm of inhibition against *K. pneumoniae.* The minimum zone 8.66 mm, of inhibition was observed in aqueous extract against *K. pneumoniae*. All the four extracts show no inhibition zones against *P. aeruginosa* as illustrated in Table 4 and Figure 3. It is well noted that Ceproflaxacene 5µg standard well discs was used as a positive control for all the samples and DMSO as negative control.

Results in Table 5 and Figure 1 indicate that there exists no cadmium at all. The concentration of iron was highest 80.43 ± 0.060 mg/kg in case of *Hyssopus officinalis.* The maximum zinc was found to the extent 67.59 ± 0.016 mg/kg in *L. usitatissimum*. Highest concentration of copper 17.70 ± 0.024 mg/kg was noted in Recipe 2. The maximum concentration of lead was noted in *Hyssopus officinalis* at 68.16 ± 0.088 mg/kg. The maximum concentration of chromium was noted in Recipe 2 having a value of 196.56 ± 0.960 mg/kg. The concentration of nickel was the highest 0.93 ± 0.016 mg/kg in Recipe 2 as plotted in Table 5 and Figure 1.

Minimum inhibitory concentrations (MIC) of Recipe 1 and Recipe 2 are shown in Table 6 and Table 7, respectively. Both of these recipes displayed minimum inhibitory concentrations (MIC) values against different bacterial strains. Recipe 1 shows MIC in methanol extract against *S. pneumoniae, P. aeruginosa, K. pneumonia* at 9000 mg/L, 10000 mg/L, and 11500 mg/L respectively. Similarly, in aqueous extracts it showed MIC only against *S. pneumoniae, K. pneumonia* at 10500 and 12000 respectively. While in chloroform extract against *S. pneumoniae, P. aeruginosa, K. pneumonia* at 9000 mg/L, 12000 mg/L and 14000 mg/L respectively.

Recipe 2 displayed MIC in methanol extract against *P. aeruginosa and K. pneumonia* at 1100mg/L and 11500 mg/L respectively. Similarly, in aqueous extracts against *P. aeruginosa and K. pneumonia* at 12000 mg/L, and 13500 mg/L, respectively. In chloroform extract against *P. aeruginosa and K. pneumonia* at 13000 mg/L and 8000 mg/L, respectively. 

## 3. Discussion

### 3.1. Comparison of Recipe 1 and Its Individual Plants

Recipe 1 contains a high amount of alkaloids (10.60%), flavonoids (28.50%) and saponins (10.90%), while its individual plant *Pistacia integerrima* contains a higher amount of alkaloids (30.00%) and flavonoids (41.20%). Alkaloids have various chemical compositions and possess a key role in drug development. It possesses antimalarial and stimulant activities of herbal plants that are also due to alkaloids [19], while the rest of the four plants in Recipe 1 contain a high concentration of flavonoids. Flavonoids are present in medicinal plants with therapeutic and antimicrobial properties [20,21]. Flavonoids also have anticancer properties and have introverted the propagation of cancer cell [22,23]. Recipe 1 also contains a high concentration of saponins as compared to its individual plants. Saponins are taught to play important role against respiratory tract infections, antitumor activity by enhancing apoptosis and gastrointestinal infections as consistent with the previous literature [24,25].

Research has proved that synthetic drugs have side effects as well as harmful in nature. Therefore, it is the need of modern age to have medicines of nontoxic nature and the medicinal plants are the only source to dissolve this dilemma [26]. Recipe 1 and their individual plants extracts as shown in Table 2 were used against three bacterial pathogens to find out the antibacterial activity. Results revealed that the methanol, ethanol and chloroform extracts of Recipe 1 show inhibition zone against *S. pneumoniae, P. aeruginosa* and *K. Pneumonia* while the aqueous extract show no inhibitory action against *P. aeruginosa.* Individual plants of Recipe 1 show inhibitory zones against all the tested bacteria except that *N. sativa* and *B. Campestris* show no zone of inhibition against *P. aeruginosa* in all extracts, *T. foenum-graecum* have no inhibition zone in methanol and chloroform extracts while *P. Integerrima* show no zone of inhibition against *P. aeruginosa* in chloroform extract. The literature survey indicates that crude extracts of some medicinal plants possess therapeutic values and can be used as useful drugs [27]. Phytochemical screening was performed for this purpose and it was proved that in some episodes the crude extracts were more active than the individual pure secondary metabolites [28].

Recipe 1 and their individual plants were analyzed for heavy metals. Cadmium (Cd) is non-essential and is very toxic in nature even at low concentration. It is the main cause of hyperactivity and slow learning in small children [29]. The accumulation of cadmium in individuals may damage the liver and kidneys. According to the World Health Organization (WHO), the maximum permissible limit of Cd is 0.3 mg/kg in medicinal plants [30,31]. Results indicate that the concentration of Cd is below the detection limit in all plants and its Recipe 1. Nickel (Ni) is an essential element for all the living organisms [32], although Ni is required in a very small quantity for an individual, as it mostly exists in the pancreas and are responsible for the production of insulin. Its deficiency below the permissible level causes disorders of the liver [33]. In a minute quantity, Ni is essential for the formation of red blood cells (RBCS). Above the permissible level, it become toxic and causes loss of vision, heart failure, weight loss and skin irritation [34]. According to WHO, the maximum permissible limit of nickel in medicinal plants is 1.5 mg/kg, while its daily recommendation for humans is 1 mg/day [30,31]. As evident from the results, that concentration was the highest at 0.69 mg/kg in the case of *Nigella sativa* and in Recipe 1, the Ni concentration is below the detectable limit. Iron (Fe) is an essential element and is an essential component of hemoglobin. It helps in the oxidation of protein, fat and carbohydrates to manage the body weight, which is a key factor in diabetes. It has a key role in an electron and in oxygen transfer in an individual body; a low level can cause myocardial infection, nosebleeds and gastrointestinal infection [35]. According to WHO, the maximum permissible limit of Fe in medicinal plants is 20 mg/kg, while its daily requirement is 10 to 28 mg/day [30,31]. The concentration of Fe was the highest 80.43 mg/kg in case of *Brassica campestris* and in Recipe 1, it was found 47.22 mg/kg. Lead is non-essential trace heavy metal having no functions in both the animals as well as in plants. High concentration of lead causes oxidative stress, colic, anemia, brain damage, headache, and central nervous system disorder [36]. It accumulates in liver, kidney and spleen through air (15%), food (65%) and water (20%). According to WHO, the maximum permissible limit of Pb in medicinal plant is 10 mg/kg [30,31]. The maximum value for Pb content was noted at *Pistacia integerrima* 74.46 mg/kg. The concentration of Pb for Recipe 1 was 53.22 mg/kg. Zinc (Zn) is essential trace heavy metal which plays an important role in various processes including brain development, bone formation, normal growth, wound healing and behavioral response. Zinc’s deficiency in a diabetic can affect the sense of smell and touch [37]. It plays an important role in protein and DNA synthesis. It is an important element of different enzymes and regulates many structural and catalytic functions [38]. According to WHO, the maximum permissible limit of Zn in medicinal plant is 50 mg/kg, while its daily requirement in food is 11 mg/kg [30,39]. The maximum Zn was found to be 76.98 mg/kg in Recipe 1, while its minimum concentration 6.66 mg/kg was noted in *Pistacia integerrima*. Chromium (Cr) is very essential for the metabolism of glucose, cholesterol and fats. Its concentration above the permissible level is toxic in nature. The toxicity of Cr intake may appear in form of stomach ulcers, skin rashes, kidney damage, and nose irritation. Its deficiency may lead to elevated body fat and disturbance in proteins, lipids and glucose metabolism [40]. According to WHO the maximum permissible level of chromium in medicinal plant is 1.5 mg/kg and its daily requirement is 0.2 mg [30,31]. The highest amount of chromium was noted in *Brassica campestris* 200mg/kg and in Recipe 1 was 199mg/kg. Copper (Cu) is essential metal for usual growth and development as well as for many enzymatic activities. The concentration of copper above the permissible level causes skin and hair discoloration [41]. Copper is concentrated mainly in the brain and kidney of an individual. Its deficiency causes anemia and Wilson’s disease [42]. According to WHO, in medicinal plants, the maximum permissible of Cu is 10 mg/kg, while its daily requirement in food is 2–3 mg/day [30]. Highest level of Cu content 16.77 mg/kg and 16.62 mg/kg were noted for *Nigella sativa* and Recipe 1, respectively.

### 3.2. Comparison of Recipe 2 and Its Individual Plants 

Recipe 2 and all of its individual plants contains alkaloids, flavonoids and saponins but *Ephedra vulgaris* (dry branches) contains highest contents of alkaloids, flavonoids and saponins. Recipe 2 has high concentration of these phytochemicals as compared to *Linum usitatissimum* and *Hyssopus officinalis*, but less than the *Ephedra vulgaris.*

*Ephedra vulgaris* also called *Ephedra gerardiana* and represents the provincial flower of the Baluchistan province of Pakistan. It is locally known as Soma kalpa. Plants of the genus *Ephedra*, including *E. sinica* and others, which has been traditionally used by indigenous people for a variety of medicinal purposes, including treatment of asthma, hay fever, and the common cold [43].

Recipe 2 and their individual plants extracts were used against three bacterial pathogens to find out the antibacterial activity. The results obtained revealed that all the extracts of *Ephedra vulgaris* show inhibition zones against *S. pneumoniae, P. aeruginosa* and *K. pneumoniae.* The aqueous and chloroform extracts show no inhibitory zone against *P. aeruginosa. Linum usitatissimum, Hyssopus officinalis* and Recipe 2 extracts also showed a very large zone of inhibition against *S. pneumonia* and *K. pneumonia* but showed no inhibition zone against *P. aeruginosa.*

As evident from the results, the concentration of Cd is below the detection limit in Recipe 2 and its plants. Ni concentration was the highest 0.93 mg/kg in the case of Recipe 2 and the lowest 0.54 mg/kg in the case of *Linum usitatissimum*. Above the permissible level, it is toxic and causes heart failure, loss of vision, loss of body weight and skin irritation [44]. The concentration of Fe was the highest 299.79 mg/kg in case of *Hyssopus officinalis* followed by Recipe 2, where the observed value was 121.11 mg/kg. The maximum value for Pb content was 68.16 mg/kg noted for *Hyssopus officinalis*, which is beyond the permissible limit of WHO, which is dangerous and cause different physiological disorder and diseases. This high quantity might be due to the obtained samples were expected to be collected from the potentially polluted industrial, vehicle and population density area as consistent with the reported literature [40,45]. The concentration of Zn was the highest 67.59 mg/kg in case of *Linum usitatissimum* followed and in Recipe 2 (60.81 mg/kg). Highest level of Cu content 17.7 mg/kg was noted in Recipe 2 followed by *Linum usitatissimum* 16.17 mg/kg. The Cr concentration was highest 196.56 mg/kg in Recipe 2 and lowest value of 182.9 mg/kg in *Linum usitatissimum.* Chromium (Cr) is essential element and its intake above permissible level is carcinogenic and leading to lung cancer. Its deficiency may cause other health-related problems like body fat and disturbance in proteins, lipids and glucose metabolism [46].

The MIC of Recipe 1 and Recipe 2 were determined by using broth dilution method against *S. pneumoniae, P. aeruginosa, K. pneumonia* as shown in Table 6 and Table 7, respectively. Both these two recipes presented MIC against all the tested bacterial strains.

## 4. Materials and Methods 

### 4.1. Plant Parts Collection and Identification

Seven selected medicinal plant parts i.e., *Nigella sativa* (seeds), *Trigonellafoenum-graecum* (seeds), *Brassica campestris* (seeds), *Pistacia integerrima* (galls), *Linum usitatissimum* (seeds), *Hyssopus officinalis* (flowers) and *Ephedra vulgaris* (dry branches) were obtained from the local herbal markets (Hakeems davakhanas) of District Karak and District Kohat, Khyber Pakhtunkhwa. Plants were used for experimentation after their identification by an expert botanist of the Department of Botany at Kohat University of Science and Technology in Kohat, Pakistan.

### 4.2. Plants Parts Grinding and Preparation of Herbal Recipes 

The collected medicinal plants parts were initially washed using tap water, dried and then sliced into tiny pieces separately. Each part was mashed into powder form separately with the help of mortar and pestle and powdered samples were safe in dirt-free, separate closed-glass containers for further use [47]. Two recipes were prepared from seven plants according to the hakim description [48,49]. Recipe 1 is the combination of four plants i.e., *Nigella sativa* (seeds), *Brassica campestris* (seeds), *Trigonellafoenum-graecum* (seeds) and *Pistacia integerrima* (galls) mixed with 1:1:2:4, respectively. It is used for colds, bronchitis, cough, asthma and gastrointestinal infections. Recipe 2 is the combination of three plants i.e., *Linum usitatissimum* (seeds), *Hyssopus officinalis* (flowers) and *Ephedra vulgaris* (dry branches) mixed at a 1:1:1 ratio, respectively, which is mainly used for bronchitis, colds and cough.

### 4.3. Qualitative Phytochemical Screening

Specific standard methods were used for the qualitative phytochemical screening of medicinal plant parts and their recipes. Alkaloids, flavonoids, tannins and saponins were detected by Tyler [50] and Harborne [51] method.

### 4.4. Quantitative Phytochemical Screening

Harborne [51] and Obadoni [52] methods were used for the quantitative phytochemical analysis to determine the alkaloids. Flavonoids were determined by Mattila and Boham [53,54] method, while Obadoni [53] method is used for saponins.

#### 4.4.1. Alkaloids Determinations

Five grams of each plant sample was stirred with mixture of 200 mL of 10% acetic acid in ethanol, covered and allowed to stand for 4 h. After filtration, the extracts were concentrated on a water bath to ¼ of the original volume. Concentrated ammonium hydroxide was added drop wise to the extract until the precipitation appeared, washed with dilute ammonium hydroxide and then filtered. The residue obtained is the alkaloid, dried and weighed [51,52].

#### 4.4.2. Flavonoids Determination

Ten grams of the plant sample was extracted repeatedly with 100 mL of 80% aqueous methanol at room temperature. The whole solution was filtered through Whatman filter paper No. 42 (125 mm). The filtrate was later transferred into a crucible and evaporated till dryness over a water bath and weighed to a constant weight [53,54].

#### 4.4.3. Saponins Determination

Twenty grams of plant sample was dispersed in 200 mL of 20% ethanol. The suspension was heated over a hot water bath for 4 h with continuous stirring at about 55 °C. The mixture was filtered and the residue re-extracted with another 200 mL of 20% ethanol. The combined extracts were reduced to 40 mL over water bath at about 90 °C. The concentrate was transferred into a 250 mL separating funnel and 20 mL of diethyl ether was added and shaken vigorously. The aqueous layer was recovered while the ether layer was discarded. The purification process was repeated. 60 mL of normal butanol extracts were washed twice with 10 mL of 5% aqueous sodium chloride. The remaining solution was heated in a water bath. After evaporation the sample were dried in the oven into a constant weight. The saponins contents were calculated in percentage [52].

#### 4.4.4. Antibacterial Activity of Medicinal Plants and Their Recipes

Pure cultures of three bacterial strains e.g., *Streptococcus pneumoniae, Pseudomonas aeruginosa* and *Klebsiella pneumoniae* were obtained and selected for further experimentation. These three bacterial strains were further subcultured on nutrient agar.

Extracts of each plant part and their recipes were prepared. The powders were extracted in four solvents: methanol, ethanol, chloroform and aqua. The mixtures were kept at room temperature for two weeks. Whatman filters paper was used for the filtration of plant materials. Rotary evaporation technique was followed to obtain semisolid extract from the filtrate [53,55]. Three bacterial strains, i.e., *Streptococcus pneumoniae, Pseudomonas aeruginosa* and *Klebsiella pneumoniae,* were used to observe the antibacterial activity. The agar well disc diffusion method was adopted for the evaluation of antibacterial activity [56]. In general, all the equipments were autoclaved and sterilized before use. Then 15 mL of the media was poured in each Petri plate and kept for cooling. The bacterial strains were applied on the Petri dishes by using sterilized cotton swab. After that, by using the sterilized Cork borer of 6 mm diameter of each Petri plate was punched into five wells for DMSO, distilled water, chloroform, ethanol and methanol crude extracts, respectively. After that, prepared the stock solution of all crude extract in DMSO each of 30mg/mL. Now each well was filled with 100 µl stock solutions except one which was filled with DMSO as a negative control. The standard disc of Ceproflaxacene (5 µg) was used as positive control. All the process was performed in the laminar flow hood in order to resist contamination. The plates were then incubated in the incubator at 37 °C for 24 h. At last the zones of inhibitions were measured in mm for each crude extract by using digital Vernier caliper and the obtained results were noted and recorded [57].

#### 4.4.5. Determination of Minimum Inhibitory Concentrations (MICs) by Broth Dilution Method

MIC is the lowest concentration (in mg/L) of the antimicrobial agent that prevents visible growth of a microorganism under defined conditions. Broth dilution techniques (micro dilution) were used to determine the minimal inhibitory concentration (MIC) of antimicrobial agents, including antibiotics that kill (bactericidal activity) or inhibit the growth (bacteriostatic activity) of bacteria. Broth dilution uses liquid growth medium containing geometrically increasing concentrations (typically a twofold dilution series) of the antimicrobial agent, which is inoculated with a defined number of bacterial cells. The antibacterial agents were dissolved in DMSO. After incubation, the presence of turbidity or sediment indicates growth of the organism [58,59].

#### 4.4.6. Heavy Metals Analysis of Medicinal Plants and Their Recipes

All the sample solutions were analyzed by flame atomic absorption spectrophotometer (model Perkin Elmer 400 using nitrous oxide (N_2_O) acetylene flame). About 2700 °C, temperature was produced in the ignition chamber and provided enhanced reducing settings for the atomization of the respective heavy metal. Each sample solution was aspirated by nebulizer, converted into an aerosol, mixed with the gases of flame, and conditioned into atomic form. Only a small portion, about 5% of the total sample, was allowed to aspirate which significantly controlled interferences. All the sample solutions were analyzed for the estimation of trace heavy metals like Fe, Ni, Zn, Cu, Cr, Cd, and Pb.

### 4.5. Data Analysis

Data was analyzed and organized by means of Microsoft Excel. The entire experiments were performed in triplicate. Standard deviations as well as average zone of inhibitions were calculated by Microsoft Excel 2007. SPSS versions 16 were applied to determine the phytoconstituent activities.

## 5. Conclusions

In summary, the present study shows the medicinal importance of all the tested plants and their recipes used against different infectious diseases that has shown potent activity against disease causing microbes. Therefore, it has been concluded that these medicinal plants individually as well as in their recipes can be used against various infectious diseases with enhanced activity, especially against respiratory tract infections. This might be due to the presence of different phytochemicals, i.e., alkaloids, flavonoids and saponins, as proved by different qualitative and quantitative assays, while the heavy metals analysis were carried out by the atomic absorption spectrophotometric technique, which also ascertains the different concentrations of heavy metals. Some plant samples showed a high amount of heavy metals like iron (Fe), lead (Pb) and chromium (Cr) metals, which might be due to the samples collection from the industrially polluted area. Thus, we concluded that these plants and its recipes are the active materials used against different diseases causing bacteria. Furthermore, these plants can be used as a source of new drug development for the curing of diseases. Finally, this study may be helpful in identifying new bioactive compounds from medicinal plants used against pathogenic microbes.

## Figures and Tables

**Table 1:** Quantitative phytochemical screening of alkaloids, flavonoids and saponins of Recipe 1, Recipe 2 and their individual plants.

S.No	Recipes/Plants (Part Used)	Alkaloids (%)	Flavonoids (%)	Saponins (%)
1	Recipe 1	10.60	28.50	10.90
2	*N. sativa* (seeds)	1.30	13.00	1.15
3	*B. campestris*(seeds)	3.22	10.00	0.35
4	*T. foenum-graecum* (seeds)	3.23	13.80	2.45
5	*P. integerrima* (galls)	30.00	41.20	3.60
6	Recipe 2	7.80	12.70	1.00
7	*L. usitatissimum*(seeds)	3.21	6.10	0.65
8	*H. officinalis*(flowers)	5.00	8.10	1.05
9	*E. vulgaris* (dry branches)	10.40	24.70	2.20

**Table 2:** Antibacterial activity of Recipe 1 and its individual plant extracts against three bacterial pathogens.

Recipe/Plant (parts used)	Bacteria	Methanol Extract (mm)	Ethanol Extract (mm)	Chloroform Extract (mm)	Aqueous Extract (mm)	Cipr (mm)	DMSO (mm)
Recipe 1	*S. pneumonia*	14.23 ± 0.20	10.70 ± 0.16	14.43 ± 0.30	12.33 ± 0.28	28	-
	*P. aeruginosa*	14.70 ± 0.16	10.70 ± 0.16	12.26 ± 0.20	0	0	-
	*K. pneumoniae*	14.66 ± 0.16	16.46 ± 0.12	12.46 ± 0.16	15.50 ± 0.16	32	-
*N. sativa*	*S. pneumonia*	13.21 ± 0.16	19.40 ± 0.08	12.36 ± 0.28	20.23 ± 0.16	33	-
	*P. aeruginosa*	0	0	0	0	0	-
	*K. pneumoniae*	12.03 ± 0.16	13.20 ± 0.12	14.26 ± 0.20	14.30 ± 0.16	30	-
*B. campestris*	*S. Pneumoniae*	11.76 ± 0.12	22.40 ± 0.24	16.36 ± 0.28	16.26 ± 0.20	32	-
	*P. aeruginosa*	0	0	0	0	0	-
	*K. pneumoniae*	12.70 ± 0.16	15.46 ± 0.16	13.16 ± 0.16	12.43 ± 0.20	29	-
*T. foenum-graecum*	*S. pneumonia*	12.13 ± 0.12	12.50 ± 0.16	14.40 ± 0.29	14.03 ± 0.12	31	-
	*P. aeruginosa*	0	12.30 ± 0.16	0	0	0	-
	*K. pneumoniae*	16.70 ± 0.16	16.63 ± 0.09	11.30 ± 0.24	11.53 ± 0.28	28	-
*P. integerrima*	*S. pneumonia*	16.36 ± 0.20	16.43 ± 0.24	18.43 ± 0.36	20.06 ± 0.16	33	-
	*P. aeruginosa*	8.30 ± 0.16	9.53 ± 0.16	0	0	0	-
	*K. pneumoniae*	14.60 ± 0.24	18.56 ± 0.24	16.50 ± 0.32	19.36 ± 0.32	34	-

Note: ANOVA value (p < 0.01) for all samples.

**Table 3:** Heavy Metals in Individual Plants and Its Recipe 1.

Recipe/Plant (parts used)	Cd (mg/kg)	Fe (mg/kg)	Zn (mg/kg)	Cu (mg/kg)	Pb (mg/kg)	Cr (mg/kg)	Ni (mg/kg)
Recipe 1	BDL	47.22 ± 0.010	76.98 ± 0.019	16.62 ± 0.043	53.22 ± 0.178	199.92 ± 0.218	BDL
*Nigella sativa*	BDL	59.61 ± 0.023	68.52 ± 0.027	16.77 ± 0.009	53.79 ± 0.577	197.91 ± 0.216	0.69 ± 0.014
*Brassica campestris*	BDL	80.43 ± 0.060	53.94 ± 0.019	6.3 ± 0.019	65.1 ± 0.062	200.07 ± 0.152	0.63 ± 0.015
*Trigonellafoenum-graecum*	BDL	55.92 ± 0.023	45.24 ± 0.023	12.24 ± 0.017	52.8 ± 0.738	192.72 ± 0.316	BDL
*Pistacia integerrima*	BDL	42.03 ± 0.030	6.66 ± 0.009	5.1 ± 0.011	74.46 ± 0.165	191.97 ± 0.502	BDL

Note: Cd was observed below detection level.

**Table 4:** Antibacterial activity of Recipe 2 and its individual plant extracts against three bacterial pathogens.

Recipe/Plant (parts used)	Bacteria	Methanol Extract (mm)	Ethanol Extract (mm)	Chloroform Extract (mm)	Aqueous Extract (mm)	Cipr (mm)	DMSO (mm)
Recipe 2	*S. pneumoniae*	12.46 ± 0.32	8.70 ± 0.16	12.73 ± 0.12	8.66 ± 0.16	20	-
	*P. aeruginosa*	0	0	0	0	0	-
	*K. pneumoniae*	12.30 ± 0.16	12.50 ± 0.16	20.06 ± 0.16	10.30 ± 0.16	33	-
*L. usitatissimum* (seeds)	*S. pneumoniae*	11.23 ± 0.16	14.60 ± 0.08	12.40 ± 0.08	0	31	-
	*P. aeruginosa*	0	0	0	0	0	-
	*K. pneumoniae*	14.30 ± 0.16	9.30 ± 0.16	13.43 ± 0.16	14.2 ± 0.16	32	-
*H. officinalis* (flowers)	*S. pneumoniae*	12.46 ± 0.12	20.30 ± 0.16	14.30 ± 0.24	16.26 ± 0.20	32	-
	*P. aeruginosa*	0	0	0	0	0	-
	*K. pneumoniae*	8.56 ± 0.40	10.70 ± 0.16	10.03 ± 0.12	18.56 ± 0.24	33	-
*E. vulgaris* (dry branches)	*S. pneumoniae*	15.36 ± 0.24	15.30 ± 0.16	17.16 ± 0.16	13.26 ± 0.16	33	-
	*P. aeruginosa*	10.36 ± 0.24	8.70 ± 0.16	0	0	0	-
	*K. pneumonia*	12.70 ± 0.16	11.60 ± 0.08	12.63 ± 0.20	13.70 ± 0.16	34	-

Note: ANOVA value (p < 0.01) for all samples.

**Table 5:** Metals in herbal plants and in their Recipe 2 e.g., Cd, Ni, Fe, Pb, Zn, Cu and Cr.

Recipe/Plant (parts used)	Fe (mg/kg)	Zn (mg/kg)	Cu (mg/kg)	Pb (mg/kg)	Cr (mg/kg)	Ni (mg/kg)
Recipe 2	121.11 ± 0.680	60.81 ± 0.021	17.70 ± 0.024	65.37 ± 0.343	196.56 ± 0.960	0.93 ± 0.016
*Linum usitatissimum*	45.03 ± 0.045	67.59 ± 0.016	16.17 ± 0.039	57.93 ± 0.255	182.91 ± 0.244	0.54 ± 0.025
*Hyssopus officinalis*	299.79 ± 0.084	17.19 ± 0.013	8.82 ± 0.031	68.16 ± 0.088	188.07 ± 0.371	BDL
*Ephedra vulgaris*	117.51 ± 0.008	7.89 ± 0.004	3.33 ± 0.024	66.81 ± 0.343	188.07 ± 0.249	0.57 ± 0.003

Note: Cd was observed below detection level.

**Table 6:** Minimum inhibitory concentration (MIC) of Recipe 1 against different bacterial strains.

S. No	Bacteria	Methanol Extracts (mg/L)	Aqueous Extracts (mg/L)	Chloroform Extracts (mg/L)
1	*S. Pneumoniae*	9000	10,500	9000
2	*P. aeruginosa*	10,000	N/A	12,000
3	*K. pneumonia*	11,500	12,000	14,000

**Table 7:** Minimum inhibitory concentration (MIC) of Recipe 2 against different bacterial strains.

S. No	Bacteria	Methanol Extracts (mg/L)	Aqueous Extracts (mg/L)	Chloroform Extracts (mg/L)
1	*S. pneumoniae*	11,000	2000	13,000
2	*P. aeruginosa*	N/A	N/A	N/A
3	*K. pneumonia*	11,500	13,500	8000
